# Perioperative Optimization With Nutritional Supplements in Patients Undergoing Gastrointestinal Surgery for Cancer (PROGRESS): Protocol for a Feasibility Randomized Controlled Trial

**DOI:** 10.2196/10491

**Published:** 2018-10-31

**Authors:** Pablo Emilio Serrano, Sameer Parpia, Saeda Nair, Leyo Ruo, Marko Simunovic, Oren Levine, Emmanuelle Duceppe, Carol Rodrigues

**Affiliations:** 1 Department of Surgery McMaster University Hamilton, ON Canada; 2 Department of Oncology Ontario Clinical Oncology Group McMaster University Hamilton, ON Canada; 3 Department of Oncology McMaster University Hamilton, ON Canada; 4 Department of Health Research Methods, Evidence and Impact McMaster University Hamilton, ON Canada; 5 Department of Anesthesia McMaster University Hamilton, ON Canada; 6 Department of General Internal Medicine Centre Hospitalier de l’Université de Montréal Montreal, QC Canada; 7 Juravinski Hospital Hamilton Health Sciences Hamilton, ON Canada

**Keywords:** perioperative care, nutritional supplements, gastrointestinal cancer, gastrointestinal surgery, postoperative outcomes

## Abstract

**Background:**

Postoperative morbidity following gastrointestinal tract major surgery ranges between 40% and 60%. Malnutrition, poor protein intake, and surgery-related impairment of the immune system and its function have been associated with postoperative infections. Supplemental perioperative nutrition may improve nutrition by increasing protein intake to influence cell-mediated immunity, thereby reducing the rate of postoperative infectious complications.

**Objective:**

The primary objective of our trial is to determine the proportion of eligible patients randomized in an 18-month period. The primary feasibility outcome will be to (1) stop, main study not feasible: estimated proportion of randomized patients <40.0% (40/100); (2) continue with protocol modifications: estimated proportion of randomized patients 40.0% (40/100) to 59.0% (50/100); or (3) continue without modification: estimated proportion of randomized patients ≥60.0% (60/100). The secondary objectives are to evaluate compliance with the nutritional supplements and to estimate differences in postoperative complications, global health-related quality of life (QoL), and median length of hospital stay between the groups.

**Methods:**

This is a double-blind randomized placebo-controlled feasibility trial. The intervention comprises three nutritional supplements: a protein isolate powder (ISOlution); immunomodulation (INergy-FLD), formulated liquid diet; and carbohydrate loading (PreCovery). Patients will consume 1 serving of the protein supplement per day from the randomization time up to 6 days before surgery (30 days in total). The immunomodulation, a solution that contains arginine, protein isolate, omega-6 fatty acids, and RNA, aims to attenuate excessive inflammatory responses and to replenish nutrients. This solution will be consumed as 3 doses per day for 5 days before and after surgery. Carbohydrate loading helps to reduce the stress from surgery by decreasing insulin resistance. Patients will have 2 servings the evening before surgery and 1 serving 2-3 hours before surgery. To be eligible, patients must have a resectable gastrointestinal cancer for which an elective operation is planned. Patients will be stratified according to nutritional status. The operation should occur within 4 weeks from enrollment.

**Results:**

We expect to screen 165 eligible patients; 60.6% (100/165) of them will be randomized to either intervention or placebo. Assuming a two-sided alpha of .05, this will give us a 95% CI around the estimate of 53%-68%. A sample size of 50 per group will enable us to estimate the treatment effect and corresponding variance of the complication rate and QoL measures with adequate precision. The success is defined as the proportion of eligible patients randomized as ≥60.0% (60/100). Patients’ compliance is defined as an intake of at least 70% (41/58) sachets of the intervention volume.

**Conclusions:**

The results will help to determine the feasibility of a larger randomized controlled trial to implement a perioperative nutritional supplement program for patients undergoing gastrointestinal surgery for cancer.

**Trial Registration:**

ClinicalTrials.gov NCT03445260; https://clinicaltrials.gov/ct2/show/NCT03445260 (Archived by WebCite at http://www.webcitation.org/72CAmMzgP)

**International Registered Report Identifier (IRRID):**

PRR1-10.2196/10491

## Introduction

Despite recent advances in surgical techniques and perioperative management, postoperative morbidity, including infectious complications, following major surgery of the gastrointestinal tract remains high, ranging between 40% and 60% [1,2]. Preoperative fasting induces the body to utilize stored nutrients, thereby accelerating the release of stress hormones, exacerbating insulin resistance, delaying wound healing, increasing morbidity and mortality, and extending the length of hospital stay [3]. Additionally, surgery-related impairment of the immune system and its function have been associated with postoperative infections [4,5].

Often, patients undergoing surgery experience disorders of the immune response, which are facilitated by low caloric intake and intestinal bacteria translocation [2]. With surgical trauma, the balance between lymphocyte T helper type 1 (Th1) cells and T helper type 2 (Th2) cells is shifted more toward Th2 cells. Th1 cells secrete interferon-gamma and interleukin (IL)-2, and they induce cell-mediated immune responses, whereas Th2 cells produce IL-4, IL-10, and IL-13, providing help for humoral immune responses [1,6]. Th1 cells activate macrophages and are highly effective in clearing intracellular pathogens, whereas Th2 cells suppress cell-mediated immunity [1]. It is thought that this reported suppression of Th1 response and intensification of Th2 response, often reported in surgical patients, may be one of the factors increasing the susceptibility to infections and septic complications. The presence of any complication within the first 30 days postoperatively is the most important independent determinant of 30-day mortality and overall long-term survival [7]. It has been shown that supplemental perioperative nutrition can influence cell-mediated immunity, the Th1:Th2 differentiation ratio and can help reduce the rate of infectious complications after surgery, thereby improving the rate of long-term survival [1].

Oral supplements or immunonutrition that are considered to boost the immune system in this protocol are defined as a solution that contains nutrients such as arginine and omega fatty acids. Arginine deficiency after surgical stress was first reported over 30 years ago, and recent studies have demonstrated that the perioperative use of an arginine-supplemented diet has the ability to decrease the rate of postoperative infections [8]. Arginine is an amino acid involved in tissue repair and wound healing. It is an essential metabolic substrate for immune cells and required for normal lymphocyte function [9]. In addition, omega fatty acids, such as n-6 and n-3, are derived from fish oil, and they have been shown to attenuate the production of inflammatory compounds and ultimately reduce the cytotoxicity of inflammatory cells [10]. In a phase II trial, docosahexaenoic acid (DHA) supplementation was shown to increase the time to disease progression and overall survival in patients receiving adjuvant chemotherapy for metastatic breast cancer. The median time to disease progression was 3.5 months in the low DHA group and 8.7 months in the high DHA group (*P*=.02). The median overall survival was 18 months in the low DHA group and 34 months in the high DHA group (*P*=.007) [11]. Each element works toward improving the immune response against cancer through modulation of excessive inflammatory responses and replenishing depleted nutrients when the body is in a state of stress, such as surgery [11-14].

Along with impaired immune function, surgery increases the release of stress hormones, pushing the body into a catabolic state. These hormones induce the hepatic production of glucose by gluconeogenesis and glycogenolysis and reduce glucose uptake in peripheral tissues, thus leading to postoperative hyperglycemia and a state of and insulin resistance [3]. This period of resistance can be sustained for 3-4 weeks after surgery and is associated with delayed wound healing, increased morbidity, mortality, and prolonged hospital stay. The degree of postoperative insulin resistance is significantly affected by the metabolic status of the patient at the time of surgical stress. The common practice of fasting patients from the evening before surgery is used to avoid pulmonary aspiration after elective surgery. However, there is no evidence to support this [15]. In fact, preoperative fasting increases metabolic stress, hyperglycemia, and insulin resistance [16]. The preoperative protocol has since been updated to allow patients to consume clear fluids until 2 hours before surgery. Preoperative carbohydrate-rich drinks have the ability to achieve a rise in insulin to levels known to change metabolism from a fasted to fed state and reduce postoperative insulin resistance by up to 50% as well as reduce protein loss and improve muscle function [17]. In order to sustain this anabolic state and reduce the degree of postoperative resistance, it is recommended to consume 100 g of complex carbohydrates the evening before surgery and 50 g up to 2 hours before surgery; this practice has been endorsed by several anesthesiology societies [18].

For patients with gastrointestinal cancer, insufficient protein intake, insulin resistance, and postoperative immobility increase the risk of impaired immune function [19]. Insufficient protein intake also results in slower recovery, prolonging hospital stay and immobility [19]. Nutritional depletion is a major determinant of the development of postoperative complications. It is associated with changes in body composition, tissue wasting, and impaired organ function, which lead to impaired immune and muscle function. Thus, nutritionally depleted patients are at higher risk of infectious complications, and a direct relationship with increased operative mortality independent of the type of surgery has been observed [20]. Patients with gastrointestinal cancers typically experience malnutrition, significant weight loss, and reduced food intake. Thus, optimizing nutritional status both before and after surgery by meeting protein requirements creates an opportunity to reduce patients’ postoperative complications [21]. Whey protein substrates have great potential to be used effectively to support postsurgery anabolism. Whey proteins of high quality have proved to be effective in modulating muscle protein synthesis and are a convenient way to supplement protein needs in malnourished patients [22]. Whey proteins also have immunomodulating properties, including biosynthesis of the antioxidant glutathione, which could attenuate the catabolic effects of surgery and spare protein [23]. Albumin, muscle function tests, immunological status, and weight loss have been used to show the correlation between nutrition depletion and postoperative morbidity and mortality.

It has been proposed that perioperative immunomodulation, carbohydrate loading, and increased protein intake may have the potential to decrease overall complications, improve patients’ quality of life (QoL), improve disease-free and overall survival, and reduce overall health care cost by decreasing the length of hospital stay and readmissions [24,25].

There is some evidence that each intervention works separately through different mechanisms of action. Therefore, we believe that a combination of the 3 interventions could have an additive effect. We propose to carry out a study to establish the feasibility of a randomized controlled trial comparing perioperative nutritional supplements with placebo, targeted at reducing the postoperative complication rate in patients undergoing gastrointestinal surgery for cancer (NCT03445260). The secondary objectives are to evaluate compliance with the intervention (nutritional supplements) and to estimate differences in postoperative complications and the comprehensive complication index (CCI), which is a scoring system to measure postoperative complications for each patient [26]. The results of this study will provide us with the necessary information to plan a larger multicenter randomized controlled trial. Set criteria for success will be clearly outlined in this proposal to determine whether it is feasible to move forward with a larger trial. The phase III randomized trial would compare the proportion of postoperative complications, patients’ QoL, time to initiation of adjuvant chemotherapy, and its effect on disease-free and overall survival as well as costs to the health care system between groups.

This study will focus on supplementing patients’ perioperative care with 3 different products administered around the time of surgery: ISOlution, INergy-FLD, and PreCovery. ISOlution is a neutral-tasting protein isolate supplement that contains no fillers, sweeteners, or artificial flavors. It comprises a mixture of whey protein isolate and lecithin and is added to foods and drinks without altering their texture or taste due to its neutral consistency. ISOlution has 93% protein purity, a digestible indispensable amino acid score of 1.09, and 14.3 g of leucine per 100 g of protein, which is an amino acid that has been shown to have an important role in enhancing the anabolic effects of protein. ISOlution can also be administered as a tube feed. INergy-FLD is an immune-modulating solution that contains whey protein isolate, refined fish oil with omega-6 fatty acids, and antioxidants such as vitamins A, C, D, and E and has an elevated amino acid concentration with 4.2 g of L-arginine per serving. It has a natural citrus flavor, low sugar content, and a trace of lactose. Therefore, it can be tolerated by patients who are lactose intolerant. PreCovery contains 50 g of complex carbohydrates per serving, with a 12.5% carbohydrate concentration and easily mixes with water. It has no added sugar, a natural citrus flavor, and maintains a low osmolality of 114 mOsmol/kgH_2_ O, which promotes digestion, gastric emptying, and water absorption. These products are to be used as supplements and not as a sole source of nutrition.

The general objective of this study is to improve the postoperative outcomes in patients undergoing any type of gastrointestinal cancer surgery. The primary study objective is to determine the proportion of eligible patients randomized in an 18-month period. The secondary objectives are (1) to determine the proportion of enrolled patients who complete the perioperative nutritional support program (see Secondary Outcomes); (2) to estimate the difference in the proportion of patients experiencing postoperative complications between the intervention and placebo groups at 90 days following the index surgery; (3) to estimate the difference in the CCI (a scoring system to measure postoperative complications) between the 2 groups at 90 days following index surgery; (4) to estimate the difference in the global health-related QoL between groups at 90 days following index surgery; and (5) to estimate the median length of hospital stay for each group.

## Methods

### Recruitment

This is a single-center placebo-controlled randomized feasibility study comparing the intervention of perioperative nutritional supplements (immunomodulation, carbohydrate loading, and protein isolate) with an identical placebo for each solution in patients with gastrointestinal cancer undergoing surgery. Study participants will be recruited from the Juravinski Hospital and Cancer Centre (JHCC), Ontario, Canada. Participants aged ≥18 years with a resectable type of gastrointestinal cancer (eg, gallbladder, liver, pancreas, stomach, small intestine, colon, or rectum cancer) for which an elective operation is planned (resection vs palliative procedure) will be eligible. Patients with distant metastasis and patients who are lactose intolerant are also eligible because the amount of lactose in ISOlution and PreCovery is minimal.

Patients will be excluded from the study if they have type 1 diabetes, malabsorption syndrome (eg, chronic pancreatitis), organ failure (liver or kidney), galactosemia, end-stage liver disease with a Child-Pugh score of ≥B [27], end-stage renal disease (stages 3 and 4 with a glomerular filtration rate of 30-59 mL/min per 1.73 m^2^ for stage 3 and 15-29 mL/min per 1.73 m^2^ for stage 4 [28]), inflammatory diseases such as rheumatoid arthritis, systemic lupus erythematosus, Crohn’s disease and ulcerative colitis, galactosemia, ongoing infection, or poorly controlled type 2 diabetes mellitus (ie, patients who have high blood glucose of 12.0-14.9 mmol/L or higher on a regular basis). Patients who cannot tolerate oral intake (eg, gastric outlet obstruction or delayed gastric emptying), patients currently on steroids, and female patients who are pregnant or lactating will not be included in this study.

In summary, our standard surgical techniques include the following for each specific procedure:

1. Liver resection: ~60% (~50/80) are performed laparoscopically.
2. Pancreaticoduodenectomy: all are performed open; there is no routine placement of feeding jejunostomy; ~70% (~50/70) are performed with the classic approach, while the rest are performed preserving the pylorus.
3. Distal pancreatectomies: ~80% (~16/20) are performed laparoscopically, either spleen-preserving or not, depending on tumor location.
4. Rectal resection: routine total mesorectal excision, ~30% (~10/30) are performed laparoscopically.
5. Colon resections: high ligation of the colic vessels, ~40% (~30/70) are laparoscopic.
6. Gastrectomy: all are performed open with a modified D2 lymphadenectomy (no splenectomy).

### Randomization

Randomization will be conducted centrally by the Ontario Clinical Oncology Group (OCOG). Study participants will be identified by screening all patients scheduled for surgery at JHCC. Eligibility must be confirmed by the treating physician or designated prior to enrollment. After confirmation and documentation of written informed consent, patients will be randomized by accessing OCOG’s Web-based Interactive Registration and Randomization System. Randomization will be performed according to a prescribed computer-generated schedule.

### Stratification

Stratification will be employed prior to enrollment to ensure a balance between treatment arms for a factor that may influence the primary outcome, including the nutritional status of the patient. Prior to randomization, patients’ overall risk of malnutrition will be determined using the Malnutrition Universal Screening Tool (MUST) [29]. Patients will be stratified by risk status as low risk of malnutrition (MUST score of 0) versus medium and high risk of malnutrition (MUST score of 1 or 2).

### Blinding

This is a double-blinded study design. All research personnel and study participants will be blinded throughout the trial. To ensure all investigators and research personnel remain blinded, the company producing the nutritional supplements and placebo will be responsible for providing the Pharmacy Research Support Services (PReSS) and information technology (IT) technician with the allocation information specific to each lot number. All prepackaged kits for preoperative intake and postoperative intake will be sent directly to PReSS. The kits received by PReSS will include a lot number that will correspond to either the intervention or placebo; however, as mentioned above, the only personnel aware of the lot numbers and associated randomization allocation will be the independent pharmacy lead, pharmacy technicians at PReSS, and the independent IT technician responsible for the randomization sequence. To ensure all research personnel remain blinded to the patients’ allocation, PReSS will remove the lot number from each kit. PReSS will also be responsible for the accountability of the supplements throughout the study.

After entering the patient information into the Interactive Registration and Randomization System, PReSS will be responsible for dispensing the appropriate kit number and ensuring the lot number has been blinded from the patient and the research personnel. The individual packets within the kits will be labeled from “30 days before surgery” to “5 days after surgery.”

### Study Agents

#### Nutritional Supplements and Placebo

Patients undergoing gastrointestinal cancer surgery will either receive perioperative nutritional supplements or placebo (Multimedia Appendix 1). The operation should occur within 4 weeks from study enrollment. Upon assessment, patients will have a consultation with their physician where standard recommendations on nutrition prior to their surgery will be provided. This consultation will happen in the B3 wing or the Surgical Oncology Clinics of JHCC. Immediately following randomization (same day), patients will receive the intervention or placebo. The intervention consists of the following 3 different solutions.

#### Protein Isolate Powder (ISOlution)

This will be consumed by the patient to increase muscle protein synthesis and achieve the recommended per meal protein intake prior to surgery as well as after surgery. Each serving delivers 20 g of protein stirred into a minimum of 250 mL of liquid or soft foods. Patients will be asked to consume 1 serving per day (20 g of protein powder in total per day) from the time of randomization up to 6 days before surgery (up to 30 days in total).

#### Immunomodulation-Formulated Liquid Diet (INergy-FLD)

In preparation for surgery, patients will consume an immune-modulating formula containing various ingredients, including arginine, protein isolate, omega-6 fatty acids, and RNA, aimed to attenuate excessive inflammatory responses without being immunosuppressive and to replenish nutrients that are depleted in a state of stress (ie, surgery), thereby enhancing the recovery process [1]. The volume of this solution is 250 mL per dose (51 g of powder reconstituted in 250 mL of cold water). Patients will be asked to consume 3 doses per day for 5 days prior to surgery and 5 days following surgery.

#### Carbohydrate Loading (PreCovery)

On the day of surgery, a carbohydrate-rich solution will be consumed by the patient to reduce the stress of surgery, reduce insulin resistance, and accelerate recovery. The volume of this solution is 400 mL per dose (55 g of powder reconstituted in 400 mL of cold water). It contains 50 g of complex carbohydrates at a 12.5% carbohydrate concentration, including 2 g of glucides or sugars. Patients will be administered 2 servings the evening before surgery and 1 serving 2-3 hours before anesthesia.

### Feeding Tube Administration

The intervention or placebo could be administered orally or via alternate enteral feed such as gastrostomy or jejunostomy feeding tubes. ISOlution and INergy-FLD can be administered enterally via oral intake or a tube feed. This procedure is only administered if oral intake cannot be tolerated. For tube feeding administration of ISOlution, mix 1-3 servings into 60-1120 mL of water and stir until completely dissolved; infuse via syringe down the feeding tube; and flush the tube with 30-60 mL of water before and after administration. To administer INergy-FLD via tube feed, mix 1 serving of INergy-FLD with 250 mL of water and stir until completely dissolved and infuse via syringe down the feeding tube; flush the tube as necessary (30-60 mL water). ISOlution and INergy-FLD contain lactose at 0.03 and 0.06 g per serving, respectively. A single threshold of lactose for lactose intolerant subjects cannot be determined. However, the trace amount of lactose found in ISOlution and INergy-FLD is not predicted to cause adverse effects.

### Placebo

There will be a placebo control for each of the solutions administered to patients in the intervention arm. The placebo will look exactly as the intervention externally (package) and internally (white powder). Each placebo is composed of a collagen-based filler with exactly the same taste and texture as the intervention. The placebo will be produced and provided by the start-up company Enhanced Medical Nutrition.

### Administration

Following randomization, patients will receive blinded packages of either the intervention or the placebo, which will be administered by the PReSS, located in the A4 wing at JHCC. The patients will be responsible for administering their own supplements while at home.

Patients will be asked to consume 1 serving of 20 g of protein isolate powder mixed in 250 mL of liquid or soft foods every day from the date of randomization to up to 6 days before surgery. Additionally, they will be asked to consume 3 servings of 51 g of an immune-modulating formula mixed in 250 mL of cold water for 5 days before and after surgery as well as 2 servings of 55 g of carbohydrate-rich powder mixed in 400 mL of cold water the evening before surgery and 1 serving 2-3 hours before anesthesia. Administration of the carbohydrate loading substance will be modified for patients following the Enhanced Recovery After Surgery (ERAS) Protocol. All study participants who are also taking part in the ERAS Protocol will be asked not to take the carbohydrate loading supplement as per the ERAS Protocol and will be asked to take the carbohydrate loading substance (or placebo) provided for this study. Routinely at our center, patients undergoing colorectal surgery are enrolled in an ERAS Protocol that encourages drinking fluids on postoperative day 1. Patients undergoing hepatobiliary surgery (either pancreas or liver surgery) are not included in an ERAS pathway; however, they are also encouraged to start a diet usually composed of liquids on day 1 after surgery. Patients undergoing gastric surgery are kept nothing per mouth for 7 days. However, they are started on tube feeds on day 1 after surgery.

At this point, patients will fill out a daily compliance diary from the date of randomization until up to 5 days after surgery. This compliance diary will be transcribed into a preoperative compliance case report form (CRF) that includes the number of packets the patient took every day, the volume that was ingested, the days it was ingested for, and the reasons patients were not compliant. While in the hospital, the nurses will be responsible for administration, as PReSS will facilitate the administration of the supplements (INergy-FLD) by adding them to the nursing care pathway of the patients. Compliance during this time will be obtained from the nurses’ log, which includes the amount of solution the patient ingested during each administration and the days the patient ingested the solution. If for some reason, the patients are unable to tolerate an oral diet (postoperative complications such as delayed gastric emptying, ileus or bowel obstruction, etc), the nurse will contact the principal investigator of the study and the order to suspend the administration of the solution will be assessed for each patient. This will be noted in the nurses’ chart and transcribed to the postoperative compliance CRF. Patients will continue to be part of the study.

### Patient Follow-up

**Figure 1:**
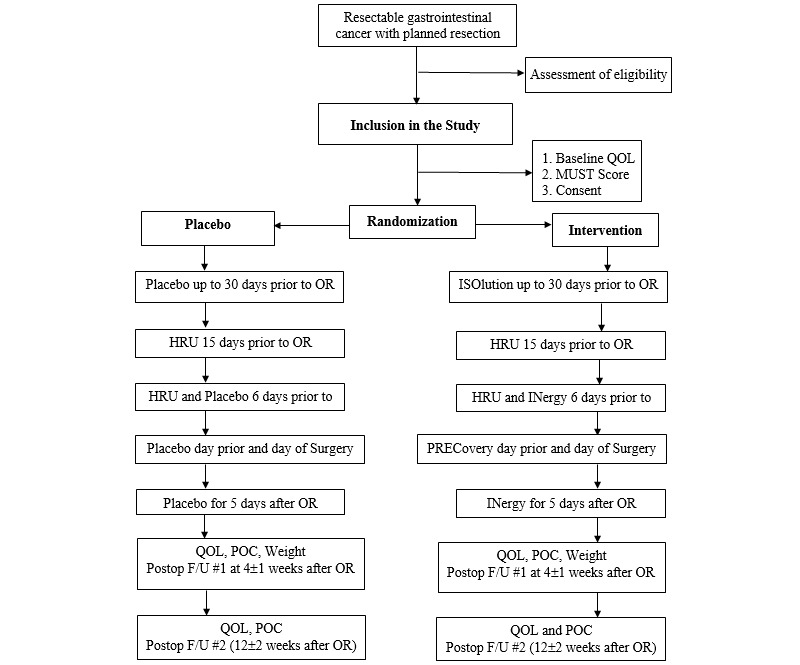
Study schema. F/U: follow-up; HRU: health resource utilization assessment; MUST: Malnutrition Universal Screening Tool; OR: operating room (index surgery); POC: postoperative complication assessment; QoL: Quality of Life Assessment.

At the baseline visit, patients will have a complete history and physical examination recorded including height, current weight, and weight 6 months prior. Patients will complete a baseline QoL assessment during their clinic visit (Multimedia Appendix 1 and Figure 1). Patients will be asked to complete a health resource utilization form at 15 and 6 days before surgery (Multimedia Appendix 1). Immediately after surgery, patients will be followed on a daily basis during their hospital stay to collect postoperative complications and compliance data and length of hospital stay (Multimedia Appendix 1). Following discharge from the hospital, patients will then be assessed at their first postoperative clinic visit 4 weeks (±1 week) following the index surgery. The second postoperative follow-up will happen at 12 weeks (±2 weeks) following surgery. At each assessment, a physical examination will be completed. A QoL questionnaire will be given to patients to complete while they wait in the surgical clinics. They will also be asked to complete a health resource utilization form at each assessment. Mortality will be recorded 90 days after surgery.

### Data Management

In addition to physical examination and QoL data, a research assistant will gather postoperative complication data from patients’ charts or electronic medical records and transcribe it on to CRFs, which will be updated daily while the patient is in the hospital. Before surgery, a compliance diary will be provided to patients to evaluate their adherence. Compliance data will also be collected from the nurses’ charts while the patient is in the hospital, with a specific CRF designed to record the volume of the supplement the patient has taken after surgery. The feasibility CRF will include reasons for two different processes: (1) reasons for not consenting and (2) reasons for not randomizing if the patient consented. Data will be stored in a secured electronic database at OCOG.

### Sample Size and Feasibility

Currently, there are 200 gastrointestinal resections performed annually for gastrointestinal cancer at JHCC (300 over an 18-month period). We expect at least 55.0% (165/300) patients to be eligible for the study. Additionally, we expect that 60.6% (100/165) eligible patients will be randomized to either intervention or placebo. Assuming a two-sided alpha of .05, this will give us a 95% CI around the estimate of 53.0%-68.0%. A sample size of 100 (50 per group) will enable us to estimate the treatment effect and corresponding variance of the complication rate, CCI, and QoL measures with adequate precision. Given the number of patients that undergo surgery at JHCC and the number of patients eligible for the study, we believe we can complete accrual to the study in 18-21 months. Given that the follow-up is 3 months, we expect the study to be finalized within 21-24 months of commencement.

### Statistical Analysis

Patient baseline characteristics and demographics by treatment group will be presented using descriptive statistics. The proportion of eligible patients randomized and its corresponding 95% CI will be calculated using the Wilson method. The criterion for success of this study is defined as the proportion of eligible patients randomized as ≥60.0% (60/100). If the estimated proportion is <40.0% (40/100), the trial will be considered not feasible. If the proportion is between 40.0% (40/100) and 59.0% (59/100), the trial will be considered feasible, with modifications to improve enrollment.

The difference in the proportions of any postoperative complication between groups and its corresponding 95% CI will be calculated using the Wilson method. The proportion of patients who are compliant with study therapy and 95% CI will also be calculated. Differences in compliance between treatment groups will be described. Good compliance will be defined as consumption of ≥70% (at least 41/58) sachets of the study intervention before and after surgery. If the compliance is ≥70% (at least 41/58 sachets), there will be no exploratory analysis to evaluate the reasons for noncompliance. Compliance of <70% (40/58 sachets or less) will be considered poor, and reasons for noncompliance will be further explored (eg, problems with the distribution or administration of the supplements, bad taste, side effects, inability to tolerate oral intake, etc) These reasons will be clearly stated in the compliance CRFs.

The mean (SD) of the CCI at 90 days from the index surgery will be estimated for each group, and the mean difference between groups will be estimated with its corresponding 95% CI. QoL scores will be summarized using means and corresponding SDs. The mean difference in the QoL scores between groups at 1 and 3 months will be estimated using linear models adjusting for baseline QoL scores.

### Ethical Considerations

The study will be performed in accordance with the recommendations for guiding physicians in biomedical research involving human patients by the 18^th^ World Medical Assembly, Helsinki, Finland, 1964. There are no perceived risks with respect to carbohydrate loading, protein solution, and immunomodulation solution as they are unlikely to interfere with cancer. The supplements being used are classified as food products, and as a result, there are no requirements in the Health Canada Food and Drug Regulations for this study. The Hamilton Integrated Research Ethics Board has approved the study protocol and documents prior to initiation. Written informed consent will be obtained from all patients prior to enrollment in compliance with International Conference of Harmonization and Good Clinical Practice guidelines and the REB.

## Results

### Primary Outcome

The primary outcome for each eligible patient will be defined as being randomized to intervention or placebo. The primary feasibility outcome will be one of the following: (1) stop, main study not feasible: estimated proportion of randomized patients <40.0% (40/100); (2) continue with protocol modifications: estimated proportion of randomized patients between 40.0% (40/100) and 59.0% (59/100); or (3) continue without modification: estimated proportion of randomized patients ≥60.0% (60/100).

### Secondary Outcomes

The secondary outcomes of the study will be defined as follows:

1. Compliance: intake of at least 70% (41/58) sachets of study intervention volume.
2. Overall complications: occurrence of any postoperative complication (major or minor) from surgery following each patient’s hospital stay and up to 90 days from the initial operation. Occurrence of any postoperative infections will also be calculated.
3. CCI at 90 days from the index surgery will be determined for each patient. This index can be calculated for each patient using the CCI Web-based calculator [26,30] following the grading of each postoperative complication according to the Clavien-Dindo classification [31].
4. QoL: The global health-related QoL at baseline, 1 month, and 3 months following randomization will be measured using the European Organization for Research and Treatment of Cancer Quality of life Questionnaire [32,33] instrument and the Functional Assessment of Cancer Therapy-General scale [34].
5. Length of hospital stay will be determined for each patient.

Compliance with the intervention is a secondary objective and will be taken into consideration for the success of its feasibility, as modifications to the protocol may be needed if compliance is poor. Compliance will be measured as the percentage volume of prescribed study intervention consumed, which will be measured by a patient diary in the preoperative period and nurses’ charts in the postoperative period. Postoperative complications (major or minor) will be determined following each patient’s hospital stay and up to 90 days from the initial operation. This is classified according to Clavien-Dindo classification [26,31,35].

### Adjudication

An adjudication committee consisting of 2 experts in the field will review each patient’s complications using deidentified source documents including discharge summaries, operative reports, interventional radiology reports, imaging reports, microbiology reports, and physician hospital and clinic progress notes as well as consultation notes from other physicians. The first adjudicator will review each complication, confirming that all reported complications are accurate, not duplicated, and appropriately classified. Whenever there is agreement with the site-reported outcome, then the outcome is considered confirmed. If there is a disagreement between the site and the first adjudicator, the second adjudicator will review that particular file. Any disagreement will be resolved by consensus, either by agreeing with the site or with the first adjudicator. Variables recorded include the length of hospital stay, blood work results, microbiology data, operating room time, estimated blood loss, number of blood transfusions during surgery and the hospital stay, and reoperations or readmissions. Each complication must be supported by source documents. The outcome assessment will follow the strict criteria set by Clavien-Dindo classification [26,31,35] and the CCI [26,31]. The outcome assessors will undergo adjudication training.

### Adverse Events

The study will be conducted according to the International Conference of Harmonization and Good Clinical Practice consolidated guidelines. Currently, there are no foreseeable risks in administering nutritional supplements to patients who meet the eligibility criteria. Nutritional supplements are safe when used by adults as instructed [36,37].

**Table 1:** The study timeline.

Planned completion date	Study goals
December 2017-September 2018	Attain Initial Research Ethics Board Approval, receive initial shipment of the Nutritional Supplements and Placebo, and submit Research Ethics Board Amendments for approval required prior to recruitment
October 2018	Begin recruiting patients
July 2018	Have 50.0% (50/100) of participants enrolled in the study
April 2020	Complete study enrollment. Have 100% (100/100) of participants enrolled in the study
May-September 2020	Complete final follow-up visits
October-December 2020	Complete final statistical analysis and begin preparing manuscript
December 2020	Have final manuscript completed and ready for publication

If an adverse event (AE) occurs during the study and is deemed related to the administration of the study treatment, this will be reported using version 4.0 of the Common Terminology Criteria for Adverse Events (Multimedia Appendix 2) [38]. This data will be collected from the first administration of treatment until the last study visit.

The investigational food products are ISOlution (whey protein isolate), INergy-FLD (immunonutrition), and PreCovery (carbohydrate loading). AEs will be considered related to study product if they are deemed to be related specifically to the administration of ISOlution, INergy-FLD, or PreCovery.

Worsening of gastrointestinal cancer is expected and therefore will not be considered an AE for the purpose of this study. Deaths due to gastrointestinal cancer are outcome events and will not be reported as AEs.

### Scientific Reporting and Publication

The Steering Committee is responsible for the scientific reporting, publishing, and presentation of the study results. All investigators participating in this study must agree to delegate the primary publication or presentation responsibility to the Steering Committee. Any other publication or presentation related to the study and the results by any investigator or participant must receive prior approval from the Steering Committee. No other publication or presentation is allowed before the primary publication or presentation by the Steering Committee. Authorship will be determined by the Steering Committee. The information developed during the conduct of this study is considered confidential.

The timeline for the study can be found in Table 1. We obtained research ethics approval from our local REB in December 2017. We aim to recruit 50 (100) patients of the patients by July 2019. The estimated study completion date is December 2020.

## Discussion

Many patients in Ontario undergo surgery for gastrointestinal cancer each year. These surgeries are often associated with postoperative morbidity and infectious complications. Therefore, it is crucial to actively take steps to aid in recovery and improve patient QoL through perioperative optimization. Despite the debate on the role of perioperative nutritional supplements in improving postsurgical outcomes, we feel there is enough clinical interest in the surgical community to support a well-designed, randomized controlled trial addressing this question. The study will provide quality preliminary evidence for perioperative nutritional supplements and determine the feasibility of recruitment, randomization, and compliance, thereby providing the necessary information to design a phase III trial if the results of the study are favorable.

## Appendix

Multimedia Appendix 1 Schedule of study procedures.

Multimedia Appendix 2 Common terminology criteria for adverse events (CTCAE) v4.0.

Multimedia Appendix 3 Peer-review report.

## Abbreviations

AE: adverse event
CCI: Comprehensive Complication Index
CRF: case report form
DHA: docosahexaenoic acid
ERAS: Enhanced Recovery After Surgery
FLD: formulated liquid diet
IL: interleukin
IT: information technology
JHCC: Juravinski Hospital and Cancer Centre
MUST: Malnutrition Universal Screening Tool
OCOG: Ontario Clinical Oncology Group
PReSS: Pharmacy Research and Support Services
QoL: quality of life
REB: Research Ethics Board
Th: T helper
