# Supplementary material for: Perioperative Optimization With Nutritional Supplements in Patients Undergoing Gastrointestinal Surgery for Cancer (PROGRESS): Protocol for a Feasibility Randomized Controlled Trial
Source: JMIR Res Protoc. 2018 Oct 31;7(10):e10491. doi: 10.2196/10491 (PMC6257881; doi:10.2196/10491)
Supplement: Multimedia Appendix 1 [file resprot_v7i10e10491_app1.pdf]

Multimedia Appendix 1. Schedule of study procedures.

Study procedure	Screening	Baseline	15 days prior to OR	6 days prior to OR	5 days prior to OR	Day prior to OR	Day of OR	5 days post OR	Follow-up 1	Follow-up 2	End of study
		30 days prior to OR[a]							4 weeks (±1 week) from the date of OR	12 weeks (±2 weeks) from the date of OR	
Review list of gastrointestinal surgeries	X										
Eligibility form	X										
Enrolment log	X										
Informed consent process	X										
Baseline case report form		X									
Quality of life (European Organization for Research and Treatment of Cancer Quality of life Questionnaire)		X							X	X	
Malnutrition Universal Screening Tool Score		X									
Randomization		X									

Patient Compliance Diary		X	X	X	X	X	X	X	X	X	
ISOlution or placebo		X	X	X							
INergy or placebo					X			X			
Precovery Or placebo						X	X				
Postoperative follow-up form #1 (includes ECOG[b])									X		
Postoperative follow-up form #1 (includes ECOG)									X		
Postoperative follow-up form #1 (includes ECOG)									X		
Major complication form									X	X	
Health resource utilization form			X	X					X	X	
Deidentify data and send all study documents											X

[a]OR: operating room (index surgery).
[b]ECOG: Eastern Cooperative Oncology Group.
