# Supplementary material for: Perioperative Optimization With Nutritional Supplements in Patients Undergoing Gastrointestinal Surgery for Cancer (PROGRESS): Protocol for a Feasibility Randomized Controlled Trial
Source: JMIR Res Protoc. 2018 Oct 31;7(10):e10491. doi: 10.2196/10491 (PMC6257881; doi:10.2196/10491)
Supplement: Multimedia Appendix 2 [file resprot_v7i10e10491_app2.pdf]

APPENDIX 1 – Common Terminology Criteria for Adverse Events (CTCAE) v4.0

The NCI Common Terminology Criteria for Adverse Events is a descriptive terminology which can be utilized for Adverse Event (AE) reporting. A grading (severity) scale is provided for each AE term.

Components and Organization

System Organ Class (SOC), the highest level of the MedDRA hierarchy, is identified by anatomical or physiological system, etiology, or purpose (e.g., SOC Investigations for laboratory test results). CTCAE terms are grouped by MedDRA Primary SOCs. Within each SOC, AEs are listed and accompanied by descriptions of severity (Grade).

CTCAE Terms

An Adverse Event (AE) is any unfavorable and unintended sign (including an abnormal laboratory finding), symptom, or disease temporally associated with the use of a medical treatment or procedure that may or may not be considered related to the medical treatment or procedure. An AE is a term that is a unique representation of a specific event used for medical documentation and scientific analyses. Each CTCAE v4.0 term is a MedDRA LLT (Lowest Level Term).

Definitions

A brief definition is provided to clarify the meaning of each AE term.

Grades

Grade refers to the severity of the AE. The CTCAE displays Grades 1 through 5 with unique clinical descriptions of severity for each AE based on this general guideline:

Grade 1 Mild; asymptomatic or mild symptoms; clinical or diagnostic observations only; intervention not indicated.

Grade 2 Moderate; minimal, local or non-invasive intervention indicated; limiting age-appropriate instrumental ADL*.

Grade 3 Severe or medically significant but not immediately life-threatening; hospitalization or prolongation of hospitalization indicated; disabling; limiting self-care ADL**.

Grade 4 Life-threatening consequences; urgent intervention indicated.

Grade 5 Death related to AE.

Note: A Semi-colon indicates ‘or’ within the description of the grade. A single dash (-) indicates a grade is not available. Not all Grades are appropriate for all AEs. Therefore, some AEs are listed with fewer than five options for Grade selection.

Grade 5

Grade 5 (Death) is not appropriate for some AEs and therefore is not an option.

Activities of Daily Living (ADL)

*Instrumental ADL refer to preparing meals, shopping for groceries or clothes, using the telephone, managing money, etc.

**Self-care ADL refer to bathing, dressing and undressing, feeding self, using the toilet, taking medications, and not bedridden.
