# Supplementary material for: Perioperative Optimization With Nutritional Supplements in Patients Undergoing Gastrointestinal Surgery for Cancer (PROGRESS): Protocol for a Feasibility Randomized Controlled Trial
Source: JMIR Res Protoc. 2018 Oct 31;7(10):e10491. doi: 10.2196/10491 (PMC6257881; doi:10.2196/10491)
Supplement: Multimedia Appendix 3 [file resprot_v7i10e10491_app3.pdf]

Juravinski Hospital and Cancer Centre Foundation

December 18, 2017

Dr. Pablo Serrano
Juravinski Hospital
B3-161
711 Concession Street
Hamilton, ON L8V 1C3

Dear Dr. Serrano:

Congratulations! We are pleased to inform you that your research project submission entitled **"PeRioperative Optimization with Nutritional Supplements in Patients undergoing GasRointEStinal Surgery for Cancer (PROGRESS), a pilot feasibility study"** has been reviewed by the Grants and Distribution Review Panel and a decision has been made to approve funding from the Juravinski Hospital and Cancer Centre Foundation in the amount of **$99,992.70.**

Please find the attached summary of reviewers' comments on your proposal. While reviewers' comments are generally positive, there are several areas in your proposal that could be strengthened. We strongly encourage you to review these comments carefully and make changes to your study protocol.

In addition, any overlap in additional funding received for this proposal should be reported to Gwen Flynn at the Foundation, and may result in reduction of the award.

Please sign and return the attached "**Terms of Funding Agreement**" to the Foundation office. As outlined in the Agreement, a final report must be submitted within 6 months of the completion of the research project. All information can be sent by internal hospital mail to:

Gwen Flynn
Hamilton Health Sciences Foundation
40 Wellington Street North
Hamilton, ON L8N 3M8 **or** Email – flynngwen@hhsc.ca

Once the Foundation has received the Terms of Funding Agreement, funds will be transferred into an account in your research project name with the JCC Finance Office. These funds will be transferred in four installments – two installments of 25% each, a third installment of 25% upon receipt of an interim progress report, and a final installment as indicated in the Terms of Funding Agreement.

For access to these funds please contact Kathy Cowan in the JCC Finance Office. Please note that if funds are not accessed within the next 12 months they will be returned to the Foundation and you must re-apply for funding approval.

For additional information or clarification, please contact Gwen Flynn at (905) 521-2100 ext. 47003

Congratulations!

Denise Bryant-Lukosius, MScN, PhD
McMaster University
Co-chair, Research Review Panel

Dr. Tim Whelan, BM, BCh
Juravinski Cancer Centre
Co-chair, Research Review Panel

KC Carruthers
VP Development
Juravinski Hospital and
Cancer Centre Foundation

cc: Dr. Ralph Meyer, President, Juravinski Hospital and Cancer Centre
Rob Forbes, Senior Financial Officer
Sandra Wilson, CFO and VP Philanthropy, Hamilton Health Sciences Foundation

Grants and Distribution
Terms of Funding Agreement

TR-185 – Pablo Serrano

"PeRioperative Optimization with Nutritional Supplements in Patients undergoing GasRointEStinal Surgery for Cancer (PROGRESS), a pilot feasibility study"

In order to receive funding, please note the following:

1. Upon receipt of this **signed document**, the Foundation will advise the JCC Finance office to activate an account in your name, specifically for this grant.

2. The Foundation will transfer the grant funding in four equal installments over the estimated duration of the research project. Based on the duration of the research project in the application, 24 months, the estimated start date of February 2016 and the estimated completion date of February 2018, the estimated payment schedule to JCC Finance will be as follows:

• First installment	January 1, 2018	$24,998.10
• Second installment	July 1, 2018	$24,998.20
**INTERIM REPORT DUE**	**October 1, 2018**	
• Third installment	January 1, 2019	$24,998.20
• Final installment	July 1, 2019	$24,998.20
**FINAL REPORT DUE**	**May 31, 2020**	

3. The Interim report is due no later than **October 1, 2018**
   - How the funds have been used to date in comparison to budget
   - Expected date of completion

4. The final installment will be transferred into your account in arch 2018 based on the above schedule.

Important Note: Failure to submit the interim project report will result in delay or refusal of your subsequent installments.

5. A final report will be due **May 31, 2020** and must be submitted to the Foundation Office within 6 months of the completion of the research project including:
   - A breakdown of how the funds were used (final financial report)
   - A summary of publications referencing the research project
   - An abstract of research outcomes

Important Note: Failure to submit a final report will affect future grant application consideration by the Foundation.

6. Funds not accessed within 12 months will be returned to the Foundation by JCC Finance and the recipient must re-apply for funding approval.

7. These funds are encumbered for this specific project and any funds, which are not used, must be reimbursed back to the Foundation Office.

8. Any publications resulting from this project must acknowledge the support of the Juravinski Hospital Cancer Centre Foundation.

December 1, 2017
Expected Start Date (mm/dd/yy)

November 30, 2020
Expected Date of Completion (mm/dd/yy)

[signature]
Signature of Principal Investigator(s)
Pablo Serrano

12/19/17
Date (mm/dd/yy)

KC Carruthers – Vice President, Development
Juravinski Hospital and Cancer Centre Foundation

Date (mm/dd/yy)

Reviewer Feedback

Grant TR-185 – Pablo Serrano "PeRioperative Optimization with Nutritional Supplements in Patients undergoing GasRointEStinal Surgery for Cancer (PROGRESS) , a pilot feasability study"

Strengths:

- Straightforward research question which can be relatively easily answered, with potential for clinical impact
- Multidisciplinary team includes both junior and very experiences members
- It is likely to be completed in the time required
- Feasibility metrics are appropriate; patient acceptability rate (i.e. % who agree to be randomized) is set at a relatively high 60%
- Appropriate and clear double blind trial design
- Rationale is clearly explained
- Compliance will be assessed with patient diaries
- Strong potential for future funding

Weaknesses:

- The background section of this grant could have been strengthened in these areas:
  - Preliminary data in the form of a systematic review and survey of the field for opinions and preferences would have made the proposal stronger
  - There have been several randomized studies of immune-nutrition in GI surgeries and several meta-analyses. Not sure how much additional information this study itself will add to the overall literature base in this study.
- The rationale for the definitive trial is missing from the scientific abstract (minor point, but an issue for further grant applications).
- The main study outcome (% of eligible patients who are randomized to study) appears to be a very modest outcome for a thematic grant which is supposed to, in itself, address important questions.
- There are 3 components to the intervention – a protein powder, a sugary powder, a fat & protein powder. It will not be possible to distinguish whether any observed effect is due to 1, 2, or all 3 of these factors.
- Similarly, as a feasibility study, the success or failure is essentially due to whether the patient takes the protein powder daily for 30 days. i.e. if a patient takes the protein powder for 30 days, but does not take the sugar powder nor the protein/fat powder, they will be considered as successfully compliant.
- Patients are to consume the protein powder for 30 days prior to surgery, yet surgery is expected to occur within 4 weeks of randomization.
- It is not clear, if this study is successful, what the primary outcome of the larger RCT would be, and whether this is a feasible or clinically relevant outcome.
- It is not clear what the treatment effect (expected) would be for the definitive trial. 50% reduction in complications? What is the baseline event rate? This information is important to determine the sample size for the definitive trial. The sample size appears to be designed for the pilot study; however the size of the pilot would be determined by the size of the definitive trial.
- A ward nutritionist will be involved in study management. However, adding a PhD prepared nutritionist with methodological research expertise in this area would be advantageous for strengthening the study protocol to address study limitations noted above.
